# Recent developments in parrot cognition: a quadrennial update

**DOI:** 10.1007/s10071-022-01733-2

**Published:** 2022-12-22

**Authors:** Theresa Rössler, Alice M. Auersperg

**Affiliations:** 1 Messerli Research Institute, University of Veterinary Medicine Vienna, Medical University Vienna, University of Vienna, Vienna, Austria; 2 Department of Cognitive Biology, University of Vienna, Vienna, Austria

**Keywords:** Parrots, Psittacines, Domain-general abilities, Physical cognition, Social cognition

## Abstract

Psittacines, along with corvids, are commonly referred to as ‘feathered apes’ due to their advanced cognitive abilities. Until rather recently, the research effort on parrot cognition was lagging behind that on corvids, however current developments show that the number of parrot studies is steadily increasing. In 2018, M. L. Lambert et al. provided a comprehensive review on the status of the most important work done so far in parrot and corvid cognition. Nevertheless, only a little more than 4 years after this publication, more than 50 new parrot studies have been published, some of them chartering completely new territory. On the 25th anniversary of Animal Cognition we think this warrants a detailed review of parrot cognition research over the last 4 years. We aim to capture recent developments and current trends in this rapidly expanding and diversifying field.

“What have you been smoking?” certainly a rather unconventional reviewer response to a grant proposal. When Irene Pepperberg started her research on verbal symbolic representation in African grey parrots (*Psittacus Erithacus*) in the late 70s, this is the kind of feedback she received from some scientists (Pepperberg 2009). Such comments amply illustrate that the study of psittacine intelligence did not garner such critical acclaim in its beginnings as it does today.

Although beloved pets for centuries, both for their imitation capabilities and their ability to form attachments to humans, parrots, or birds in general, were for a long time considered incapable of complex cognition by the scientific community. This was partially driven by the absence of similar brain structures known to be involved in mammalian cognitive processing. The early terminology of vertebrate brain segments suggested that the entire avian telencephalon consisted solely of greatly enlarged basal ganglia, thus completely lacking the equivalents to the neocortical structures of mammals that would allow for complex cognition to emerge (Reiner et al. 2004a; Jarvis et al. 2005).

This theory engendered a lack of communication between avian and mammalian neuroscientists until the beginning of the twenty-first century. To counter this problem a large group of international experts organized the Avian Brain Nomenclature Consortium (Reiner et al. 2004b), and radically changed the popular view of avian brains (Reiner et al. 2004a). Although the avian and the mammalian pallia are organized differently (nuclear versus laminar), they share similarities in neurophysiological functions that are involved in complex cognitive processes. The revised terminology acknowledges those functional analogies and facilitates comparisons of cognitive abilities (Jarvis et al. 2005).

The encephalization of two avian groups stands out in particular: parrots and corvids. Though several groups studied the cognitive abilities of corvids in the 1990s and early 2000s, widespread scientific interest in parrot cognition was lagging behind for at least a decade. One possible reason for this may have been the geographical endemic distribution of the respective study subjects, with many corvid species being relatively common in (but not restricted to) areas from which fields such as ethology, behavioral ecology, and comparative psychology originated.

Although some ‘early birds’ had dipped their toes in the waters of parrot cognition (such as Nadeszha Ladygina-Kohts, Otto Köhler, and Werner Fischel; reviewed in Auersperg and von Bayern 2019), for a long time the field was largely pioneered by Irene Pepperberg with her studies of communicative and numerical cognition of the African grey parrot Alex (abbreviation for Avian Learning EXperiment; Pepperberg 1999). Since the early 2010s (but see early work on kea e.g., Gajdon et al. 2004; Huber et al. 2001; Tebbich et al. 1996) research on parrot cognition has gained momentum and the number of studies continues to rise. In M. L. Lambert et al. (2018) published a comparison of corvid and parrot cognition, providing a comprehensive review of the current state of knowledge in psittacine research. However, with the speed at which parrot cognition research is advancing, more than 50 new studies have been published in the 4 years since M. L. Lambert´s publication. This review aims to provide an update on the rapidly accumulating cognitive studies on parrots. We will provide a brief summary of the related work from neurobiology followed by a review focused on the most recent research findings on parrot cognition published since M. L. Lambert et al. (2018). Lastly, we will discuss current trends of the field.

## Parrot brains

Differentiated nervous systems are deeply connected to complex cognition (see e.g., Iwaniuk 2017). We thus briefly review the current state of knowledge on the structure and function of parrot brains. Neurological measures found to correlated with cognitive capacity typically include brain size in relation to body mass (relative brain size; but see Smeele (2022) for a discussion on possible pitfalls), size of certain areas of the brain associated with complex cognition, as well as the number of neurons and neural connectivity in those areas (see Iwaniuk 2017 for review). For example, a recent survey found that the positive association of innovation and absolute as well as relative brain size also extends to the number of pallial neurons in birds (Sol et al. 2022).

The brain structure most (but not exclusively) associated with enhanced cognition is the forebrain—more specifically the outer mantle, called pallium, of the telencephalon (also known as cerebrum) located within the forebrain. For example, the avian nidopallium caudolaterale functionally resembles the mammalian prefrontal cortex (both specific regions in the pallia) and is fundamentally important for executive functions (Güntürkün 2005; Güntürkün et al. 2021; Güntürkün and Bugnyar 2016); the relative size of the hyperstriatum ventrale, a region in the (nido)pallium involved in multimodal integration, correlates with feeding innovations in birds (Timmermans et al. 2000). The mammalian pallium (also known as the cerebral cortex) has a layered organization whereas the avian brain retains a nuclear structure. Nevertheless, molecular organizations seem to function analogically in both mammals and birds (Jarvis et al. 2013). In fact, recent findings uncovered that the fiber architecture of the avian pallium has iteratively organized circuits similar to those of mammals. This suggests that these neuronal circuits might have evolved from an ancient microcircuit shared with mammals (Stacho et al. 2020).

The avian pallium contains considerably more neurons per unit than that of mammals, i.e., they are more densely packed. Parrots and corvids in particular reach pallial neuron counts that are comparable to primates despite their obvious differences in brain size (Olkowicz et al. 2016; Herculano-Houzel 2017). Additionally, avian neurons are remarkably energy efficient: the glucose consumption of pigeon neurons was found to be only a third of that of mammals (per neuron and minute; von Eugen et al. 2022). A new study on avian brain evolution revealed that parrots and corvids likely achieved their large, densely packed brains through different evolutionary paths (Ksepka et al. 2020). Both groups show the highest rates of brain-body evolution events within the Neoaves, resulting in relatively larger brains, but while corvids simultaneously enlarged both body and brain (with brain size increase outpacing body size increase), parrots reduced their body size. Interestingly, the most profound evolutionary shifts in avian relative brain size did not occur at the origin of flight (as one might assume due to general decrease in body size) but during the aftermath of the Cretaceous-Paleogene (K-Pg) mass extinction (Ksepka et al. 2020). Furthermore, Kverková et al. (2022) recently found that four major shifts in the evolution of amniote neuron-brain scaling may have led to high neuron counts. Firstly, increased neuron density evolved independently in early mammals and birds and subsequentially large changes in neuron-brain scaling happened convergently in core land birds (Telluraves; which include parrots and corvids) and anthropoid primates.

Multiple findings suggest neuronal specialization for vocal learning and motor control in parrots: the medial spiriform nucleus (SpM) of parrots is greatly enlarged compared to other avian species. It connects the telencephalon with the cerebellum and is believed to be integral for the deliberate control of fine motor skills and complex cognitive processes in a way that it is functionally analogous to the cortico-ponto-cerebellar pathways of mammals (Gutiérrez-Ibáñez et al. 2018). Additionally, the cerebellum (believed to play an essential role in complex motor behaviors) is more foliated in parrots, corvids, and seabirds than in other bird species (Iwaniuk et al. 2006). Parrots share a neurological core song system with hummingbirds and songbirds but have an additional, unique pathway which is involved in vocal learning (Chakraborty et al. 2015).

Another comparison with songbirds revealed that large-brained parrots have a relatively enlarged subpallium within the telencephalon. It is involved in sensory information processing, sensorimotor learning, and motor control. This finding might reflect parrots’ remarkable vocal learning abilities as well as their dexterous and flexible food and object-handling behavior (Olkowicz et al. 2016). Furthermore, a study with over 130 000 individuals across 244 parrot species showed that relative brain- and body-size correlate with longevity in parrots (Smeele et al. 2022), while Wirthlin et al. (2018) comparison with 30 other bird species (not including corvids) revealed parrot-specific changes in gene expression that are associated with cognitive abilities in humans.

There is, therefore, growing evidence that the neurological underpinnings of complex cognition in parrots are well on par with corvids and primates. Additionally, the parrot brain seems to be especially equipped for motoric dexterity and vocal learning.

## Recent cognitive research

M. L. Lambert et al.’s review (2018) highlighted many similarities between corvids and parrots although the authors caution against premature conclusions due to insufficient data. The review highlighted that psittacines have been best studied in physical cognition but understudied in the social domain. It further identified gaps in parrot research in spatial and temporal cognition (such as episodic-like memory) as well as prosociality and inequity aversion. The cognitive test that, thus far, included the largest number of tested parrot species was the string-pulling task: generally, parrots were largely successful in the basic set-up (reeling in a reward that is suspended by a string) but studies showed mixed findings in control conditions. M. L. Lambert et al. (2018) further discussed the overall lack of socio-ecological knowledge on parrots, and that studies are largely based on a handful of model species (for studies including multiple parrot species see e.g., Mettke-Hoffman et al. 2002, Krasheninnikova 2014; O´Hara et al. 2017). Furthermore, the authors noticed a trend for studies on complex physical cognition tasks while core fundamental processes such as working or spatial memory were less intensely studied. In other words, compared to primates and corvids, to some extent we have been putting the cart before the wagon.

We will focus the next section on recent psittacine research of original and peer-reviewed publications since M. L. Lambert et al. (2018). Cognitive abilities are multifaceted and often interdependent, and can therefore not easily be sorted into clear-cut categories. Nevertheless, to facilitate a structured overview in the following sections, we will divide cognitive abilities into the following categories: ‘domain-general abilities’, ‘physical cognition’, and ‘social cognition’ (see also Tables 1, 2, 3).

**Table1:** Recent psittacine research on domain-general abilities

Species	Latin name	Inhibitory control			Behavioral flexibility	Problem-solving	Planning	Reasoning			Memory	
		Delayed-gratification	Rotation task	Economical decision making/contrafreeloading				Probabilistic reasoning	Reasoning by exclusion	Working memory	Spatial memory	Aversion memory
African grey parrot	*Psittacus erithacus*	Pepperberg and Rosenberger (2022)	Brucks et al. (2022)	Krasheninnikova et al. (2018); Smith et al. (2021)				Clements et al. (2018)	Pepperberg et al. (2019)	Pailian et al. (2020)		
Blue-fronted amazon parrot	Amazona aestiva					Godinho et al. (2020)						
Blue-headed macaw	*Primolius couloni*		Brucks et al. (2022)	Krasheninnikova et al. (2018)								
Blue-throated macaw	*Ara glaucogularis*		Brucks et al. (2022)	Krasheninnikova et al. (2018)							Chow et al. (2021)	
Budgerigar	*Melopsittacus undulatus*					Chen et al. (2019); Medina-García & Wright (2021); Chen et al. (2022)						
Goffin´s cockatoo	*Cacatua goffiniana*				Bobrowicz et al. (2021)	Rössler and Mioduszewska et al. (2020)	Auersperg et al. (2017); Beinhauer et al. (2018)					
Great green macaw	*Ara ambiguus*		Brucks et al. (2022)	Krasheninnikova et al. (2018)								
Kea	*Nestor notabilis*				Laschober et al. (2021)			Bastos & Taylor (2020a,b)				McLean et al. (2022)
Sulphur-crested cockatoos	*Cacatua galerita*

**Table 2:** Recent psittacine research in the physical domain

Species	Latin name	Causal inference			Objects properties and physical entities	Tool use	
		String-pulling	Trap and tube problems	Aesop´s fable		Captivity	Wild
African grey parrot	*Psittacus erithacus*	Chaves Molina et al. (2019)			Overconversation: Cornero et al. (2020)		
Blue-throated macaw	*Ara glaucogularis*		O’Neill et al. (2019, 2021)				
Budgerigar	*Melopsittacus undulatus*				Octave equivalence: Wagner et al. (2019)		
Goffin´s cockatoo	*Cacatua goffiniana*	Wakonig et al. (2021)			Template matching: Laumer et al. (2021a) weight: P.J. Lambert et al. (2021)	Auersperg et al. (2018); Osuna-Mascaró et al. (2022)	O’Hara and Mioduszewska et al. (2021)
Great green macaw	*Ara ambiguus*		O’Neill et al. (2019, 2021)				
Greater vasa parrot	*Coracopsis vasa*	Woodley of Menie et al. (2021)					
Green-winged macaws	*Ara chloroptera*	Gaycken et al. (2019)					
Kea	*Nestor notabilis*	Bastos et al. (2021b)		Schwing et al. (2019)	Object trajectory: Bastos and Taylor (2019) virtual vs. real: Bastos et al. (2021c)	Bastos et al. (2021a)	
Peach-fronted conures	*Eupsittula aurea*	Torres Ortiz et al. (2019)

**Table 3:** Recent psittacine research in the social domain

Species	Latin name	Self-recognition	Cooperation	Prosociality	Inequity aversion	Social learning
African grey parrot	*Psittacus erithacus*			Krasheninnikova et al. (2019b); Brucks & von Bayern (2020)	Krasheninnikova et al. (2019b,c)	
Blue-headed macaw	*Primolius couloni*				Krasheninnikova et al. (2019c)	
Blue-throated macaw	*Ara glaucogularis*		Tassin de Montaigu et al. (2020)	Brucks & von Bayern (2020)	Krasheninnikova et al. (2019c)	
Goffin´s cockatoo	*Cacatua goffiniana*	van Buuren et al. (2019)		Laumer et al. (2021b)	Laumer et al. (2020)	
Great green macaw	*Ara ambiguus*				Krasheninnikova et al. (2019c)	
Kea	*Nestor notabilis*	van Buuren et al. (2019)	Schwing et al. (2020, 2021)	Heaney et al. (2020)		
Peach-fronted conures	*Eupsittula aurea*					Thomsen et al. (2021)
Sulphur-crested cockatoos	*Cacatua galerita*					Klump et al. (2021)

## Domain general

We use 'domain-general’ cognitive abilities as an umbrella term for cognitive processes that can be applied in both the social and physical domains. For example, we include executive functions and memory in this category.

### Executive functions

Executive functions are processes necessary to control and monitor one´s behavior, in order to carry out non-automated responses (reviewed in Diamond 2013). The concept of executive functions is rooted in human psychology and has been adopted by cognitive scientists in recent years (see Bobrowicz and Greiff 2022 for a review of studies on executive functions in birds). The exact definitions and categorizations are controversial (see e.g. Hofmann et al. 2012; Jurado and Rosselli 2007; Miyake et al. 2000). Here, we use the classification of Diamond (2013) for ‘core executive functions’ (inhibitory control, flexibility, and working memory) and ‘higher-level executive functions’ (problem-solving, planning, and reasoning). Core executive functions can be viewed as the basic processes upon which higher-level executive functions are built (Diamond 2013; Note that the distinction between domains varies between publications. For example, as problem-solving abilities are predominantly tested with technical problem-solving tasks rather than for social problem-solving it is often found in the physical domain.)

#### Core executive functions

##### Inhibitory control

Inhibiting a prepotent reaction to a stimulus in favor of a better outcome is an essential process for exploiting new opportunities. To test for inhibitory control, researchers usually confront their test subjects with situations in which refraining to reach for, or to consume freely available food rewards, leads to a (better) pay-off (see review in Miller et al. 2019). Parrots have previously shown mixed performance: overall, they performed poorly in the detour/cylinder task (subjects have to refrain from reaching a reward through the walls of a transparent cylinder but instead move to the open ends of the cylinder to reach in; Kabadayi et al. 2017; MacLean et al. 2014); and the revised A-not-B task (a reward is moved from cup A to cup B and subjects have to refrain from choosing the previously rewarded cup A; MacLean et al. 2014). However, it is questionable whether the detour task and the A-not-B task are suitable for testing inhibitory control (for a critical evaluation of test designs see Jelbert et al. 2016; Kabadayi et al. 2018; van Horik et al. 2018). In contrast kea (*Nestor notabilis*), Goffin´s cockatoos (*Cacatua goffiniana*), and African grey parrots showed inhibition control in delayed gratification tasks in which they had to forgo an immediate reward for a better one (for review on such tasks see Miller et al. 2019). However, they waited for markedly shorter durations for larger quantities of the same reward (Auersperg et al. 2013; Koepke et al. 2015; Schwing et al. 2017). When presented with an accumulation task (food is incrementally placed near the subject but accumulation stops when the bird starts to feed), African grey parrots only waited for a maximum of 2–3 s (Vick et al. 2010).

In a recent study, Pepperberg and Rosenberger (2022) investigated this further with an African grey parrot (named Griffin), who will reoccur in multiple studies of this review. He first learned to associate tokens with pieces of highly preferred cashew, as well as that higher quantity of tokens represented a higher number of nuts. Subsequently, he was presented with two cups, one with fewer tokens and one with more tokens (2 vs. 3, or 3 vs. 4 tokens). The cup with more tokens was then pulled out of his reach, and the other cup was shortly covered with a hand but left within his reach. This was followed by the verbal label "wait". When he touched the closer cup, he would receive the number of nuts equivalent to the tokens inside (fewer tokens), whereas when he waited for a specific duration (10 s, 40 s, 160 s, 320 s, 640 s or 900 s) he was rewarded with as many pieces of nuts as in the second, distant cup (more tokens). In contrast to most other studies (but see Koepke et al. 2015) trials were randomized over the entire experiment which meant that there was no indication of how long the delay would last. The parrot chose to wait in the majority of trials, sometimes for up to 15 min. In interspersed control trials, in which the cup with more tokens was available, he seldom waited, therefore choosing the economic decision. The authors argued that the use of tokens facilitated his ability to wait as it distanced the bird from his actual item of interest (Pepperberg and Rosenberger 2022).

A different task designed to study inhibitory control is the so-called ‘rotation task’ (Bramlett et al. 2012). It involves a disc with rotating arms which each carry a reward. One arm is near the subject while the second is out of reach but steadily moves closer. Crucially, the subject is only allowed to take one reward before the apparatus is pulled away. If the subject desires the reward on the second arm it has to control the impulse to reach for the accessible reward and instead needs to wait until the second arm moves closer. A recent study found that African grey parrots wait for longer (up to 50 s) than blue-throated macaws (*Ara glaucogularis*), blue-headed macaws (*Primolius couloni*), and great green macaws (*Ara ambiguus*) for a food reward that represented an increase in quality (Brucks et al. 2022). The birds did not wait for a lesser or equal quality reward in various control conditions. Coping behavior, particularly pacing, increased the likelihood of successfully waiting for the reward. The better performance of the African grey parrots may be associated with differences in social complexity and relative brain size between the species, however the authors caution that too little is known on both neurological measures and ecological variables for firm conclusions to be made.

The same four species as in the study described above were previously tested in an economical decision-making task (Krasheninnikova et al. 2018). Birds were confronted with a choice between an immediate food reward (of either low, medium, or high value) and a token that could be exchanged for food (again of either low, medium, or high value). In the test conditions, the available food was of lesser value than the reward gained by exchanging the token. The birds, therefore, needed to refrain from consuming a readily accessible reward in order to receive the higher value reward. All parrots maximized their payoff by exchanging the token for the high-value reward but blue-throated and blue-headed macaws did so to a lesser extent when a medium-value food reward could be gained through the exchange. Krasheninnikova et al. (2018) controlled for the possible effect of token preference by interspersing trials in which food of the same or higher value than the token was already available. All but one bird chose the food when it was of higher value, and great green and blue-throated macaws mostly choose the food when an exchange would result in the same pay-off. Interestingly though, when the food and the token were of the same value, roughly half of the African grey parrots and some of the blue-headed macaws chose the token despite it not increasing the pay-off. The authors discussed that the parrots might have developed a preference for the higher-value tokens, which possibly resulted in suboptimal choices. Moreover, the interaction with the tokens, e.g., the action of the exchange, might have been in itself rewarding (Krasheninnikova et al. 2018). A possible alternative explanation for non-economic token choices was suggested by Smith et al. (2021): contrafreeloading behavior, a phenomenon in which effort to ‘earn’ a reward is preferred over freely available food (Jensen 1963). An investigation of different forms of contrafreeloading in African grey parrots found this behavior exhibited in different contexts for different individuals (Smith et al. 2021). The parrots were confronted with a choice between two options. In the first part, rewards were placed in plastic containers – one with a lid, the other without. The readily available food (accessible without the effort of opening the lid) was either of higher, the same, or lesser value. One of the four birds showed a preference for lid opening with same-quality rewards (termed ‘classical contrafreeloading’ by the authors). All birds mostly chose the food in the closed cup when the reward was of higher value, but rarely when the reward was of lower value than the open cup. During control trials with empty cups, all birds preferred the cups with lids suggesting that the interaction with the cup was in itself rewarding. In the second part of the study, two out of five parrots preferred nuts with intact shells over shelled nuts. The study, therefore, shows that contrafreeloading behavior has a strong individual component: some birds seemed to find certain actions rewarding whereas others did not (e.g., removing the shell of a nut; Smith et al. 2021).

Note that the last two studies, though conducted to test two different processes (economic decision-making vs. contrafreeloading), share methodological similarities: in each study, subjects were offered a choice between free food and the possibility to access an alternative reward by expending effort (exchanging a token, opening a lid or shelling a nut). The freely available food was either of lesser, equal, or higher value. In both studies, African grey parrots mostly chose the economical option when the payoff was unequal but roughly half of the birds exerted effort when the payoff was equal. It seems that such tasks may put African grey parrots in a conflict between two preferences (food or ‘fun’).

In summary, there is now increasing evidence, especially in African grey parrots, for inhibitiory control. The duration an individual would wait seems to be largely dependent on task designs. For example, Pepperberg and Rosenberger (2022) argue that countering immediate food impulses by replacing the reward with a token as symbolized reward, may facilitate the ability to wait in delayed gratification tasks.

##### Flexibility

Tightly connected to inhibitory control is the ability to quickly adjust one´s behavior in reaction to changing circumstances. In a recent reversal learning experiment, kea, who have previously waited up to 160 s to exchange a less desirable food reward for a preferred reward (Schwing et al. 2017), showed highly flexible behavior (Laschober et al. 2021). Test subjects had to discriminate between two images presented on a touchscreen. Pecking at one resulted in a reward whereas choosing the other did not. At the midpoint of each session these contingencies were reversed (the rewarded stimulus became unrewarded and vice versa). As a group, the kea in this study seemed to mostly adopt a win-stay/lose-shift strategy. They tended to choose an image as long as it was rewarded and quickly switched to the other stimulus when the first stopped being rewarded. However, strong within- and between-subject variation suggests that kea could also have relied on other strategies including side biases. At times, the error pattern of some individuals suggested a reversal-estimation strategy, where subjects anticipated the point of reversal (possible ways to do so might include using time, number of trials passed, or saturation levels). Furthermore, birds that could rely on spatial as well as visual information, performed better than kea that had to base their choices on visual information alone. The authors highlight the importance of considering individual variations in cognitive studies (Laschober et al. 2021).

Not every experience an animal has, and not every memory that it can recall, is necessarily useful in a future novel context. Irrelevant or conflicting information on similar problems can hamper the expression of necessary actions (see e.g., functional fixedness; Brosnan and Hopper 2014). Bobrowicz et al. (2021) tested how conflicting experiences affected Goffin´s cockatoos’ performance in novel tool use tasks. Each bird was tested on their ability to spontaneously solve two different kinds of test tasks (‘hookset’ or ‘screwset’) and thereafter received training on slightly different tasks. These training tasks were either functionally overlapping (required a similar motor action but at a different location) or perceptually overlapping (required different motor actions but at the same location). In the non-conflict condition, the birds were only trained on the functionally overlapping task while in the conflict condition they received training in both the functionally and subsequently the perceptually overlapping (but functionally different) task. Goffins had to pass a learning criterion in the training to finally proceed to test tasks 24 h after their last experience. No bird solved the test during the first exposure or 24 h later (control). Three of the seven birds were able to solve the test task after (functionally) non-conflicting training and two out of five did so after receiving (functionally) conflicting experiences. Compared to the control condition, overall, the Goffins interacted more with the correct tool after (functionally) non-conflicting training and more with parts of the task located at the correct location after conflicting training (in which the most recent training task needed to be manipulated at the same location as the test task). Success further depended on the nature of each task (‘hookset’ or ‘screwset’). Together with the small number of successful birds and large individual variation results yield a complex picture. It seems as if the successful birds did not identify the relevant aspects of the task earlier than the unsuccessful birds but rather that they changed their behavior more flexibly (Bobrowicz et al. 2021).

##### Working memory

The last core executive function is working memory – storing and manipulating (e.g., updating) information that is no longer perceptually present (Diamond 2013). To the best of our knowledge, Pailian et al. (2020) were the first to specifically target working memory in a parrot species. They used a shell game task to compare visual working memory in Griffin (the African grey parrot) as well as human adults and children. The basic principle of the shell game is as follows: test subjects observe the experimenter hide woolen pom-poms of different colors under opaque cups. Then two cups at a time are swapped, and the test subject needs to keep track of each colored pom-pom and its location. At the end of the procedure, the subject has to indicate the location of a pom-pom of a specific color. To test for different levels of difficulty, the number of swaps varied between 0 and 4, and the set size of hidden pom-poms was increased from 2 to 4 pom-poms. The parrot showed remarkable capabilities of updating and recalling the location of each colored pom-pom and chose correctly significantly above chance in all combinations. While Griffin was on par with the performance of the humans with a set of 2 pom-poms, when 3 pom-poms were used, he outperformed 6-to-8-year-old children, and, with an increasing number of swaps, even adults. His performance decreased when more than two swaps were made within a set containing 4 cups. One possible factor explaining the decrease in correct choices was that Griffin might have switched his attention from the target cup to other cups in case the target cup was not part of the switch. In a follow-up experiment the parrot was repeatedly tested with 4 cups and 4 swaps, but now the number of times the target item was part of the swap, was systematically varied (0–4 times). If attentional switching had limited Griffin performance in the earlier experiment the researchers expected his accuracy to be highest when the cup was involved in all switches. However, his performance decreased with increasing swaps of the target item. Overall, Griffin’s limit in the shell game seemed to be related to updating information rather than to attention switching or storage related factors (e.g., rate of temporal decay; Pailian et al. 2020).

#### Higher-level executive functions

##### Problem-solving

Chen et al. (2019) investigated whether problem-solving abilities can influence mate choice in female budgerigars (*Melopsittacus undulatus*). The researchers first evaluated the amount of time each female budgie chose to spend close to either of two males. Then they trained the less preferred males on two problem-solving tasks: opening a lid (one-step action) and a box (three-step action). Thereafter, both males were confronted with the puzzles for 1 min while the females could observe them. All trained males, but none of the untrained males, solved the tasks within the given time. In a subsequent preference test, females spent more time in proximity of the previously unpreferred males than the previously preferred males. To exclude the possibility that females switched their preference solely because they saw the problem-solving birds feeding, females in a control group observed a less preferred male feeding and a preferred male not feeding. To examine whether the effect was dependent on sex of the problem-solver, in a subsequent test the birds that were observed (less preferred problem-solvers and preferred non-solvers) were females. In the following preference tests, the observer females did not switch their preferences in favor of the female problem-solvers or the feeding males. As females only changed their preference when observing males that were proficient in solving problems, the authors suggested that this showed a possible direct influence of mate-choice and therefore sexual selection on problem-solving abilities in budgerigars (Chen et al. 2019).

In a later study, Medina-García and Wright (2021) investigated this hypothesis from another perspective: does being a proficient problem-solver attract more mates and lead to more reproductive success? The researchers measured performance in four different tasks: technical problem-solving, detour reaching, seed discrimination, and spatial memory, and calculated a composite cognitive score based on their cumulative performance. Upon completion of these tasks, the birds were assigned to five mixed-sex groups with a male-biased ratio of 2:1. However, the composite cognitive score did not affect female choice or female reproductive investment. Nevertheless, males with a higher score sired more offspring (both in-pair and extra-pair). Mating with a male who performed overall better in the tasks thus had significant fitness consequences for female budgies as this resulted in more fledged nestlings (Medina-García and Wright 2021).

Another recent study on budgies investigated the link between personality and cognition (Chen et al. 2022). Here, personality was measured through breathing rate during handling stress and latency to enter a novel maze. The test subjects were then confronted with (a) an initial color discrimination task, (b) a reversal task, (c) a second color discrimination task, and (d) a technical problem-solving task. Latency to enter a novel maze did not predict performance in any of the tasks. However, birds that were breathing slower during handling learned faster in the initial discrimination task but not in the reversal or second discrimination tasks (which – apart from color—were visually identical to the first task). Furthermore, they were more successful in the novel problem-solving task and interacted more with the apparatus (a novel transparent puzzle box). The results suggest that breathing rate as a measure of personality only had an effect when the parrots were tested in tasks that have a novel physical appearance (Chen et al. 2022).

A study on Goffin´s cockatoos (*Cacatua goffiniana*) investigated whether the ‘captivity effect’ influences performance in a technical problem-solving task (Rössler and Mioduszewska et al. 2020). It has been proposed that captive animals might show increased cognitive performance due to factors such as lack of predation or increased free time (see e.g., Haslam 2013). A captivity effect would therefore predict better performance by long-term captive individuals in human-designed problem-solving tasks. After being thoroughly habituated to the basic set-up, subjects were simultaneously presented with an array of 20 problem-solving tasks, requiring different motoric actions such as pushing a button, sliding a door or pulling out a drawer. In the following test sessions, short-term captive (but otherwise wild) Goffin’s cockatoos were just as likely to solve the same number of tasks as their long-term captive counterparts when they chose to interact with the task. However, wild-caught individuals were less eager to participate in the experiment, therefore showing a difference in motivation. The authors concluded that, while life in captivity may affect motivation in such artificial experiments, there is yet no evidence for a captivity effect on the technical problem-solving *capacity* in this species (Rössler and Mioduszewska et al. 2020).

To investigated the problem-solving abilities of captive and reintroduced blue-fronted amazon parrots (*Amazona aestiva*) Godinho et al. (2020) confronted them with two tasks: a pebbles-and-seeds discrimination and a multi-access-box test. The pebbles-and-seeds task examined whether the parrots could successfully feed on seeds when mixed with pebbles of similar size (seed: pebble ratio = 35:50). The multi-access-box is commonly used to test for behavioral flexibility and problem-solving (Auersperg et al. 2011, 2012a): A reward in the center of an acrylic glass cube can be accessed through four different mechanisms—one at each side of the cube (pulling a string, opening a window, inserting a ball or pushing the reward with a stick). Once a solution is found reliably (criterion depends on specific study), this side can be blocked – forcing the individual to change to a different solution. Whereas the captive blue-fronted amazons were tested individually, the reintroduced and free-ranging birds largely approached the apparatus in pairs. In those cases, one bird would typically manipulate the apparatus while the other observed. Both groups retrieved the majority of seeds in the pebble-and-seeds task (88.16% captive birds; 86.58% reintroduced birds). Left-footed captive birds were more successful in the pebbles-and-seeds task compared to right-footed individuals but not in the multi-access task. Eleven out of fourteen captive birds found the string-pulling solution and two were also able to open the window on multiple occasions. Fifteen reintroduced amazons interacted with the apparatus and three solved the string problem at least once; one opened the window. The authors conclude that captive and reintroduced blue-fronted amazons showed similar cognitive abilities with regard to the type of problems they solved (preferentially the string; some managed to open the window; Godinho et al. 2020).

*Spontaneous innovations*: Technical problem-solving tasks are regularly used as a proxy to study innovative behavior (see Griffin and Guez 2014 for a review; but see also Tebbich et al. 2016 for a critical discussion). Spontaneous and non-experimentally triggered innovations however are challenging to observe. Anthropogenic changes to a species´ environment do, however, offer increased opportunities to observe innovative behaviors as a result of behavior modifications to meet new challenges. Such an opportunity presented itself when kea were first observed to put sticks in traps designed to catch stoats (*Mustela erminea*; stoat trapping was conducted to protect endangered ground-nesting species Hegg 2006; Tansell et al. 2016). In addition to monitoring the number of sticks found in trap boxes, Goodman et al. (2018) installed camera traps on 3 trap sites and in 51 days observed a total of 67 stick insertions or insertion attempts by kea (66 of which were at one trap-box), presumably to access the egg bait within the boxes. Probing sessions lasted up to 45 min with a mean session duration of 12 min at the site with most probing attempts. The authors assumed that visits to this site might have been made by the same individual, but inserted sticks were found on multiple trap-box sites not equipped with motion cameras. As the kea´s tempering with the traps interfered with stoat trapping efforts, the trap-box design was changed to eliminate this behavior (Goodman et al. 2018). However, despite anecdotal reports it remains unclear whether the kea ever gained any value from this activity apart from a possible intrinsic effect, and further whether the camera traps captured one individual or multiple birds.

Klump et al. (2021) came upon an opportunity when they started to observe Sulphur-crested cockatoos (*Cacatua galerita*) opening trash bins in the urbanized areas of Sydney and Wollongong regions in Australia – a behavior previously also reported in a group of kea in New Zealand in Mount Cook National Park (Gajdon et al. 2006). Similar to the kea, cockatoos pried open the lids with their beaks or grabbed the handle of the bin, and held the lid high while walking towards the hinges until the lid fell or was flipped open. Importantly, the authors had the unique opportunity to track the spread of the behavior which we will elaborate on in the ‘social domain – social transmission’ section below.

A single male kea Bruce, living at the Willowbank Wildlife Reserve in New Zealand, is facing a unique challenge: He is missing the upper part of his beak which makes tasks such as preening difficult. However, he came up with a solution for daily feather care: he collected pebbles by holding them between his tongue and lower beak and used those pebbles for preening (Bastos et al. 2021a). In a different study, individual Goffin´s cockatoos have been observed to manufacture and use sets of tools, likely as a result of individual innovative behavior rather than habitual species-wide tool use (O’Hara et al. 2021). We will elaborate more on both aforementioned examples in the ‘physical domain – tool use’ section.

To summarize recent findings, innovative problem-solving may be driven by sexual selection (Chen et al. 2019) and may increase reproductive success in budgerigars (Medina-García and Wright 2021). Less fearful budgies are better problem-solvers when presented in a novel context, whereas more lateralized blue-fronted amazon parrots are not (Godinho et al. 2020). Additionally, no captivity effect was found on the capacity to solve novel problems in Goffin´s cockatoos (Rössler and Mioduszewska et al. 2020). We further noted 4 new spontaneous innovations described in parrots in the last 4 years.

##### Planning

M. L. Lambert et al. (2018) identified a complete lack of psittacine research focusing on temporal cognition. Recent studies on planning in animals are fiercely debated (see e.g., Boeckle et al. 2020, 2021; de Mahy et al. 2021; Hampton, 2019; Kabadayi and Osvath, 2017; Osvath and Kabadayi, 2018). This debate is largely focused on more sophisticated forms of planning such as episodic foresight which requires mental imagery of a future scenario. Such studies have been attempted in corvids (Kabadayi and Osvath 2017; Boeckle et al. 2020) but are, to this day, still missing in parrots. Nevertheless, in the past 4 years, there have been first attempts to study more rudimentary forms of cued planning for the near future.

Goffin`s cockatoos have been tested for ‘safekeeping’ behaviors, i.e., whether they would keep holding on to used tools for a purpose in the immediate future (Auersperg et al. 2017). They were confronted with an apparatus that provided multiple foraging opportunities: five rewards, each of which could be accessed by using a stick tool. The apparatus was placed either 1 m or 5 cm from the ground (high/low condition) and the food was either directly edible or placed in capsules that need to be opened (easy/difficult-to-handle condition). Six out of eight Goffins did indeed keep the tools between the foraging instances, mostly by holding the tool with their foot or trapping it between the foot and the previous foraging location. Safekeeping was more frequent when the platform was high, possibly because losing the tool was more costly (retrieving a dropped tool in this condition would have meant transporting it in flight). Safekeeping also occurred more often when the food was easily accessible. As Goffins used their feet for both foraging and holding the tool during safekeeping, both actions might have been more conflicting in the condition that required greater manipulation of the food (Auersperg et al. 2017).

Another study targeted Goffin´s cockatoos’ ability to prospectively or retrospectively select between two different tools (Beinhauer et al. 2018). Each tool was only compatible with one of two different tasks. During the test, only one of the tasks was available but the birds had both tools to select from. The setup allowed them to see either the task and then the two tools (prospective condition) or the two tools and then the task (retrospective condition), but never both at the same time before being allowed to make a choice. In both conditions, each tool was located in a different compartment and once the individual entered the compartment of a specific tool the door of the other compartment was closed. In a training phase the parrots first learned the basic contingencies of the procedure (e.g., that tools are located in compartments and entering one compartment results in the other one being closed) but with transparent walls, thus with both the tools and the apparatus visible. Three out of six cockatoos successfully learned this procedure, and continued to the test. In test session the experimenter could exchange transparent walls for opaque ones in order to manipulate the visibly accessible information. All three birds were then able to choose the correct tool in the prospective (seeing the task first) condition but not in the retrospective condition (seeing the tools first). This means that they were able to recall which apparatus had been presented, base their choice on what was required, and remember the correct compartment the tool was located in. However, when subjects could only briefly see the tools, then the tools were occluded before the apparatus was shown, they did not learn to choose correctly, i.e., go to the compartment containing the correct tool (retrospective tool selection). These results show a similarity to those found in a previous study in apes (Martin-Ordas et al. 2014; but see the discussion on methodological differences). One of the possible explanations for the subjects’ failure in the retrospective task could be that only the sight of the apparatus triggers problem-solving behavior and that the tools by themselves are not perceived as tools before the apparatus has been revealed (Beinhauer et al. 2018).

##### Reasoning

The last higher-level executive function we will discuss is reasoning—the process of knowledge formation by “reaching a conclusion about something from known facts or evidence” (Merriam-Webster after Völter & Call 2017; for a philosophical discussion on inferential and non-inferential reasoning see e.g., Streumer 2007).

*Probabilistic reasoning*: The ability to weigh out odds to enable maximization in uncertain situations in birds and primates has been frequently examined using cup games or container tasks (Denison and Xu 2014; Rakoczy et al. 2014; Tecwyn et al. 2017). A recent study on Griffin the African grey parrot targeted the bird’s ability to take ratios into consideration when making a choice (Clements et al. 2018). The researchers took advantage of the bird’s ability to label a variety of different objects. The procedure was as follows: two types of materials or toys (wool, cork, paper, ring, key) were mixed in a bucket in a 3:1 ratio, i.e., if drawn at random one item had a probability of 0.75 to be sampled (‘majority item’) and the other 0.25 (‘minority item’). After each draw Griffin was asked which item was hidden in the experimenter’s hand. If he guessed correctly, he would receive a reward. This meant that minority items were occasionally rewarded as well. Whereas Griffin started with a strong preference for the majority item early in the experiment, he shifted towards a strategy of matching the probability of each item, although the optimal choice to maximize pay-off would be to stick with choosing the majority item (Clements et al. 2018). This phenomenon, called diversified probability matching, has also been observed in humans (Rubinstein 2002).

Another recent study on probabilistic reasoning was conducted with kea (Bastos & Taylor 2020a). Here the parrots had to choose between two closed hands, each holding a token that was drawn from a transparent container. Kea were able to see the containers at all times but the very tops were occluded from the outside by cardboard to hide the sampling event. Each container held two differently colored tokens. Previous to testing, the birds were trained to exchange one color of token for a reward while the other color token was not rewarded. The containers were then filled with both tokens in different ratios. In all parts of the study, one token was sampled from each jar and the parrots had to choose one of the two hands holding the tokens. Kea got 20 trials per test condition and advanced to the next condition (if performance was significantly above chance) or continued with training until criterion was met before advancing within each experiment. When describing the results, we will use ‘correct’ container for the one with the highest probability of drawing the rewarding token. Experiment 1 tested for probabilistic reasoning and was conducted in three consecutive stages. In condition 1, container A held fewer rewarding than non-rewarding tokens, meaning that the probability that the experimenter would sample a rewarding token was lower in A than in B. Three out of six kea choose the correct container significantly above chance. Condition 2 aimed to test whether the parrots used absolute frequency or relative frequency for choice. Here the same number of rewarded tokens were present in both containers, but container A additionally held 100 unrewarded tokens, and container B only had 4 unrewarded tokens. Four of the six kea choose correctly above chance. Condition 3 was implemented to test for an avoidance strategy of the unrewarding tokens (total number of rewarding vs. unrewarding for container A was 57:63 and 3:63 for jar B). All kea chose container A. Three birds chose correctly in all three conditions without further training, which lead to the conclusion that some kea take probabilities of each container into account without relying on the absolute number or by employing an avoidance strategy. Experiment 2 was implemented to examine whether the kea were able to consider physical constraints when making their choice. For this reason, a physical horizontal barrier was added so that only the upper part of the jar was available for sampling. Each container had the same amount of rewarding and unrewarding tokens but the ratio in the upper accessible part was different. Here all birds were able to make the correct decisions in condition 1 while five out of six parrots did so in condition 2 (same logic but reward ratios reversed). The last experiment tested whether kea take the personal bias of the experimenter into account. Kea first learned that one person was a biased sampler, i.e., had a preference to choose the rewarding color. The first experimenter carefully looked inside the container before choosing, implying bias. The second experimenter however deliberately looked away from the container and therefore seemed to choose randomly (see original study for details on the multi-phase training procedure; Bastos & Taylor 2020a). In test trials both experimenters drew from containers with an equal ratio of the two tokens. To optimize the likelihood of receiving a rewarding token the biased person should be preferred. Half of the kea chose the biased person, which lead the authors to conclude that they incorporated social information when making their choice. As a group, kea choose correctly in the first trials of each condition in each experiment (Bastos & Taylor 2020a). In a follow-up test published later, the authors did not find any evidence that the kea would use unintended cues from the experimenters for their choices (Bastos & Taylor 2020b).

*Reasoning by exclusion*: Imagine one is given a choice between two stimuli and the information that only one of them leads to a desired outcome (usually a reward), and it is known which of the two is the non-rewarding choice. The ability to reason by exclusion describes the logical deduction that the alternative to a non-rewarding choice has to be the rewarding choice (Call 2006). Several parrot species have been tested on this ability using various tasks: Kea (Schloegl et al. 2009; O’Hara et al. 2016), Goffin´s cockatoos (O’Hara et al. 2015a), red-tailed black cockatoos (*Calyptorhynchus banskii*; Subias et al. 2019) and African grey parrots (Mikolasch et al. 2011; Schloegl et al. 2012; Pepperberg et al. 2013). Each species has proven able to solve ‘inference by exclusion’ tasks and mostly they do not seem to solve the tasks by simply avoiding the incorrect option. However, Mody and Carey (2016) criticized that animals could have still inferred that something *might* be the correct option in contrast to concluding that it *has to be* the correct option.

Recently, Pepperberg et al. (2019) attempted to address this concern by examining the African grey parrot Griffin. He was confronted with cups on a tray that were divided by a cardboard barrier. Two experimenters sat opposite to him and before each test trial, a screen was placed between Griffin and the cups. Both experimenters then showed Griffin a piece of nut before hiding it under a cup. Subsequently, the screen was removed, one experimenter briefly lifted the empty cup, and Griffin was then allowed to make a choice. After establishing that Griffin understood the basic task requirements, the researchers tested for reasoning by exclusion: Griffin was presented with 4 cups in total – two per experimenter (i.e., per side). Experimenter 1 showed Griffin that one cup was empty. Choosing the second cup of this experimenter would therefore always be successful while choosing from the other experimenter resulted in a 50% chance of being rewarded. The parrot chose the ‘safe’ cup significantly above chance. If Griffin was merely avoiding the empty cup instead of inferring where it must be instead (cup next to empty) his choices should have been evenly distributed among the remaining three cups. A possible alternative explanation for his choices – other than reasoning by exclusion – was that he applied the following simple heuristic: ‘choose the cup next to the empty cup’. To examine this possibility, the experiment was repeated but interspersed with ‘type 1 gambling’ trials in which the experimenter showing the empty cup did not bait any of the cups (she showed her empty hands to the bird). Therefore, the only way to get a reward was to choose one of the two cups on the other side (which still had a success likelihood of 50%). This is what Griffin did in most trials. To further investigate whether these results can be explained by the avoidance of the empty side, ‘type 2 gambling’ trials were interspersed – again in the normal 4-cup routine. This time the choice next to the empty cup would result in a certain reward (experimenter baiting again) but a more highly preferred reward type was hidden on the uncertain (50% chance per cup) side. Whereas Griffin chose the certain cup in 15/16 standard 4-cup trials, he went for the ‘gambling option’ in 5 out of 8 possible trials. Overall, the results from this study suggests that Griffin really did use reasoning by exclusion to solve that task, and by doing so, also outperformed 5-year-old children tested in Mody and Carey (2016; Pepperberg et al. 2019).

### Memory

Information storage is commonly categorized into working (see section ‘working memory), short-term and long-term memory (for discussions on categorization see e.g., Cowan 2008; Diamond 2013). While short-term memory has an expiration date and is limited in the amount of information it may entail, long-term memory may exist for long periods of time, even for life in some cases (Cowan 2008).

One recent study examined whether parrots could learn and recall spatial patterns over a relatively short duration (tested daily; Chow et al. 2021) while a second study investigated food aversion over longer periods of time (up to 1 year; McLean et al. 2022).

Blue-throated macaws and great green macaws were tested in a spatial search pattern task (Chow et al. 2021). Subjects were required to open wells from a so-called ‘poke-box’, which consisted of 12 wells arranged in a grid. The wells were covered and had to be broken open by the parrots to access the food reward within. The macaws were presented with two different sets of patterns with six correct and six incorrect choices: the rewards were either hidden in the three most right and three most left wells (pattern A) or in the six central wells (pattern B). The parrots received three trials a day for three consecutive days before switching to the next pattern (which pattern was presented first differed per individual). Only one great green macaw learned both patterns above criterion, while the rest of the birds only learned pattern B. Crucially, the subjects were allowed to forage on the poke-box as long as they desired, which means that there were no negative consequences of incorrect choices. Blue-throated macaws made more errors overall than great green macaws. Great green macaws took more time to manipulate the task and stayed longer in the test chamber after opening the last well, which the authors summarized as explorative behavior. The researchers, therefore, concluded that opposing trade-off strategies (accuracy vs. speed) may exist in the two species: Blue-throated macaws were faster to open any wells but made more errors while great green macaws erred less but worked at a slower pace. A possible explanation can be found in the different ecological backgrounds of the otherwise closely related species: Great green macaws are more generalist foragers, whereas blue-throated macaws have a more specialized feeding ecology. Generalist species may profit more from explorative behavior in order to adjust to changes faster, whereas more specialized forager may profit from fast application of foraging skills (Chow et al. 2021).

In a different study (McLean et al. 2022), the long-term memory of kea was examined. When wild kea were fed pellets that resulted in gastrointestinal discomfort and nausea they kept avoiding these pellets for at least 6 months, but consumption rates increased to baseline level after a year of non-aversive experience. This finding may have important implications for conservation management strategies. Poisonous pellets are used to control invasive mammals but are consumed not only by the targeted species (Kemp et al. 2019). Aversion training for vulnerable species such as the kea could prevent collateral deaths (Cowan et al. 2016). Whether the durations reported in this study show temporal limits of memory or were a result of desensitization could not be determined (McLean et al. 2022).

### Concluding remarks on domain-general cognition research

To conclude this section, existing work on executive functions was greatly supplemented in a short time span: More species were tested with inhibitory control tasks, further manifesting previous findings that parrots are able to inhibit food consumption for considerable time spans in order to gain a higher quality food reward across multiple setups (see ‘inhibitory control’). Pepperberg and Rosenberger (2022), showed that the African grey parrot Griffin was able to also wait for up to 15 min for a larger quantity of reward, and discuss the influence of distancing the rewards from the test subjects (e.g., through the use of tokens). This may have important implications for future comparisons among species.

Flexibility has further been studied in the context of a within-session reversal learning task, in which kea mostly applied a win-stay/loose-shift strategy. As previous reversal learning tests for parrots used a different procedure (reversal after reaching a criterion; see Gossette et al. 1966; Gossette 1968; O’Hara et al. 2015b) future work is needed for direct comparisons.

While recent years have given us first glimpses of parrots securing benefits for the immediate future (Auersperg et al. 2017; Beinhauer et al. 2018), there have yet been no attempts to investigate more sophisticated forms of future planning. Several new setups have been employed to test the effects of problem-solving on mate choice, reproductive success, lateralization, and the effect of rearing environment on problem-solving (see ‘problem-solving’). Similarly, innovative setups were used to test probabilistic reasoning with very promising results (Clements et al. 2018; Bastos & Taylor 2020b). Furthermore, research on reasoning by exclusion with strict controls has further confirmed that this ability is present in African grey parrots (Pepperberg et al. 2019). Finally, throughout the past 4 years, several coincidental observations of innovative behavior (outside an artificial/experimental context) were systematically recorded, leading to important insights (Klump et al. 2021; O’Hara et al. 2021; Bastos et al. 2021a). We are optimistic that the recent focus on parrot cognition will enable more such opportunities.

## Physical cognition

Even though psittacines represent a highly diverse order (nearly 400 species) they share several distinct phenotypic characteristics with which they experience their physical world: zygodactyl feet (two digits facing forward and two facing backward; Botelho et al. 2014; Carril et al. 2021), equipped with mechanoreceptors, make them excellent climbers and facilitate haptic object manipulation. Parrots also have strong curved beaks and a muscular and highly versatile tongue (Demery et al. 2011; Homberger 2017). They are known for their playfulness and curiosity as well as their tendency to haptically explore their physical surroundings – largely by utilizing their sensitive tongues in combination with their beaks (see e.g., Auersperg et al. 2014a, b; O’Hara and Auersperg 2017). Like some other birds, their beak contains a dense cluster of mechanoreceptors—the so-called bill tip organ. In contrast to e.g., ducks and geese, it is not embedded in the bone but located in the hard keratin structure (rhamphotheca) along the inside of the curve of the beak (Demery et al. 2011; du Toit et al. 2020). This suggests an adaption to intraoral food processing and facilitates tactile information processing of objects held within the beak (Demery et al. 2011; Mioduszewska et al. 2022).

Physical cognition research targets the animals’ use and understanding of their physical environment. It includes, for example, causal reasoning, means-end understanding, and object permanence.

### Causal reasoning

There are a few experiments that have become benchmark paradigms to examine multiple aspects of causality such as the string-pulling task (for review see Jacobs and Osvath 2015), the trap tube task (e.g., Liedtke et al. 2011) or the Aesop´s fable task (for review see Jelbert et al. 2015). Through variations studies have asked slightly different questions regarding the mechanisms underlying performance during these tasks. For easier comprehension, we will divide the following section first into task categories, followed by experiments concerning the use of object properties and specific physical entities. Lastly, we will discuss work on tool use in a separate section.

#### String-pulling tasks

The iconic string-pulling task is used to test for a wide range of abilities such as causal reasoning or understanding contact and connections, and means-end understanding (see e.g., Jacobs and Osvath 2015 for a comprehensive review). In its most basic form, a reward is attached to a string and the test subject can retrieve the reward only by repeatedly pulling the string towards itself. Variations of this task include crossed strings, broken strings, parallel strings, visually-occluded rewards on strings, etc., and depend on the specific question of the study. The string-pulling paradigm is by far the test conducted on most parrot species (see especially Krasheninnikova et al. 2013; Krasheninnikova 2013, 2014; Krasheninnikova and Schneider 2014). Overall, psittacines solved the basic version of the task (pulling a string to access the reward or choosing the correct of two parallel hanging strings). Some species failed in the crossed condition that is designed to control for choices based on proximity while others showed an understanding of connectivity in the task (for a comprehensive review see Jacobs and Osvath 2015).

Five new studies on string-pulling in parrots have been published in the past 4 years. Gaycken et al. (2019) tested green-winged macaws (*Ara chloroptera*) to examine whether experience gained in one variation of the string-pulling task can be transferred to another. To do so they implemented a condition in which the string had to be pulled down instead of up. The string was wound around a pivot that was attached at a position higher than the bird and therefore required a downward pulling movement. One group experienced the classical ‘pull-up’ task before being tested with the pull-down condition, while the second group was only presented with the pull-down task. None of the birds in either group successfully retrieved the reward in the pull-down condition (although the group experienced in pulling up used significantly more unsuccessful pull-down actions than the inexperienced group). The authors argued that the perceptual feedback of the reward moving closer with each action reinforced their pulling behavior, and may explain this behavior rather than a transferable understanding of the task contingencies. In both cases, the reward moves closer to the participant when the correct behavior is exhibited but the visual feedback in the pull-down test might arguably be more difficult to trace (Gaycken et al. 2019).

When kea were presented with a choice between two horizontally arranged coiled strings – one connected to a reward and one broken – they failed to choose correctly (Bastos et al. 2021b). To test whether this was due to a lack of experience with perceptual-motor feedback they further received ten trials of the standard vertical single string task. All subjects succeeded in this task. However, in a subsequent second test with the horizontal set-up, they again failed. Thus perceptual-motor feedback experience was insufficient to solve the horizontal connectivity task.

A group of three juvenile African grey parrots received multiple variations of the paradigm with two strings: parallel, slanted, crossed, connected vs. disconnected, and visually accessible vs. non-visible reward (Chaves Molina et al. 2019). All birds showed strong side or color preferences and did not perform above chance in initial choices. However, two parrots often changed to the other string after an incorrect first choice. Again the results hint towards a significant role of perceptual feedback through the movement of the reward.

In contrast to the findings above, six out of nine Goffin’s cockatoos solved the task in a condition controlling for perceptual feedback: the rewarded string was coiled up on the floor and therefore the reward itself only started to move after multiple initial pulls (Wakonig et al. 2021). Furthermore, they mostly chose at random when rewards were attached to two strings that differed in length, which suggests that they did not base their choice on proximity to the reward. However, all Goffin’s mostly chose the incorrect string in the crossed condition. Given the set-up, it is unclear whether visual impairment (the strings were not fully visible from the pulling position) or an inability to understand the connectivity explains the failure.

Peach-fronted conures (*Eupsittula aurea*) also failed at a crossed stings condition but with full visual access, while mostly being able to solve other often-tested variations (parallel, slanted and broken string; Torres Ortiz et al. 2019). In the pulley condition, similar to that used on the green-winged macaws mentioned earlier, the two males succeeded while the two females stopped interacting with the strings. After demonstrations by the experimenters on how to solve the single pulley task, all four birds were tested in a pulley task with multiple strings and performed significantly above chance on a group level. However, they failed a broken pulley task that tested their understanding of connectivity. Interestingly the two previously successful individuals that solved both the broken string and the pulley with multiple strings were not able to solve the broken pulley. As most birds were able to choose the correct string in the ‘standard’ broken string condition requiring upwards pulling, an understanding of connectivity can be suggested. The failure in the broken pulley condition may therefore be attributed to the overall complexity of the task (Note: all individuals were relatively young, 18–23 months old at testing; Torres Ortiz et al. 2019).

A new anecdotal report of a single greater vasa parrot (*Coracopsis vasa*) spontaneously and repeatedly solving a string-pulling task led Woodley of Menie et al. (2021) to re-analyze parts of the data published by Krasheninnikova (2014). Based on the average performance of 14 parrot species in 5 different string-pulling variants, they extracted a score for each species (see original article for details on statistical methodology). This score was found to be highest in greater vasa parrots and spectacled parrotlets (*Forpus conspicillatus*), and was highly correlated with the fission–fusion intensity of each species as previously found in Krasheninnikova (2014; Woodley of Menie et al. 2021).

These recent studies on string-pulling behavior showed pulling by a downward movement over a pivot was more difficult for the parrots than the classical pull-up movement (Gaycken et al. 2019; Torres Ortiz et al. 2019). Direct perceptual feedback seems essential for most subjects tested, however, kea were not able to use this experience in the horizontal connectivity task (Bastos et al. 2021b). Goffin´s cockatoos solved the coiled condition in which only delayed perceptual-motor feedback was received (Wakonig et al. 2021). Overall, the parrots failed either the crossed and/or broken conditions and the results are thus in line with earlier studies on parrots which have so far reported mixed findings (for a concise list see Wakonig et al. 2021).

#### Trap and tube problems

Another benchmark test paradigm for causal cognition is the so-called trap tube task. Therein, subjects have to retrieve a reward from inside a horizontal tube with a tool or a prearranged pulling device while avoiding losing the reward to a trap inside the tube (Visalberghi et al. 1995). Solving variations of such a task has proven difficult for many species (see e.g., reviewed in Schloegl and Fischer 2017), and parrots have so far failed most attempts (Liedtke et al. 2011). Simpler heuristics, such as side biases, might guide performance in these tasks and overshadow a possible expression of an understanding of causality. O’Neill et al. (2019) recently modified the test apparatus to reduce the influence of such heuristics. Instead of a tube, they used a rectangular platform (= ‘table’), eliminated the use of tools so that the subjects could directly interact with a reward container, and avoided transparent material. Instead, the table was surrounded by wire mesh. The parrots therefore needed to reach inside the wire with their beak and push the container forward in incremental steps from one end to the other. The table had two parallel lanes along which the parrots could move the reward container.

In the first part of the study, two tables were used with both having one lane with a trap into which the reward could fall and be lost. For table A, the second lane was equipped with a white board over which the container with the reward could slide and thus be moved toward the open end. Table B had a hole in the second lane. If the container was moved over the hole it would fall onto the table and within reach of the subject. None of the parrots preferred one lane over the other, i.e., they lost the reward to the trap in roughly half of the trials. In the next stage of the experiment, each bird was trained to succeed in either table A or table B (individuals randomly assigned). Then tests were repeated with the table the subject was not trained on and subsequently each parrot was confronted with two new tables. Table C had a non-functional trap covered with a block in one lane and an open trap in the other. However, both ends of the table were blocked. This meant the only way to solve this task was by moving the reward into the ‘open trap’ whereby it could fall on the table and become accessible to the birds. For table D, the opposite was true. For this table, the ‘open trap’ was directly positioned on the table and the reward could not land below the apparatus. In contrast to table C, the route with the block inside the trap would lead to an open end from which the reward could be retrieved. Three of the nine great green macaws and seven out of nine blue-throated macaws were able to learn to solve table A and/or table B but only one parrot solved table C, and none solved table D (however, one out of five great green macaws, tested 1 year later, was able to solve table D but not table C). The authors concluded that although both great green macaws and blue-throated macaws were able to learn the contingencies of the task, they did so without causal understanding of its physical mechanisms (O’Neill et al. 2019).

The same great green and blue-throated macaws were further presented with the challenge of a multi-stone construction problem (O’Neill et al. 2021; modified after Visalberghi and Trinca 1989). A reward was placed in the center of a transparent tube and a selection of stones was provided in proximity to the apparatus. To get the reward out of the tube the parrots had to place multiple stones one after the other into the same end of the tube, building a chain of stones. This sequential stone insertion only provided perceptual feedback of the reward moving after the 3^rd^ stone was added. All birds failed to spontaneously solve the task in the given time. They were then confronted with an end-state presentation: a functional stick was pre-inserted into the tube and could be used to push the reward. After this experience, they were again tested in the original task. One out of eight blue-throated macaws solved the test task with multiple stones after this training. For the remaining birds, training continued by presenting a short tube with the reward inside. Thus the birds only needed to insert one stone to push the reward. If they still failed to solve this tasks, one stone was pre-inserted into the short tube and upon success previous training was repeated (short tube but no pre-inserted stone). Two more birds succeeded in the original test after experience with the short tube.

In transfer trials, the three successful birds were confronted with two sets of two tubes. In the first set, only one of the tubes was baited. In the second set, both were baited but one tube was closed at one end, rendering it unsolvable. Two birds failed with the first set suggesting non-goal-directed behavior. The third bird (a blue-throated macaw) succeeded in choosing the correct tube with bait in the first set but then failed to account for the barrier at the end of one tube in the second set. Again, the birds showed no functional understanding of the apparatus and the previous successes of birds in test sessions could be explained by a side bias in combination with explorative behavior (all tested birds did insert stones at some point during the experiment; see Auersperg et al. 2014a, b for a studies on playful object insertions). The only individual tested in the second transfer task did not consider the physical barrier. Whether he did not attend to it or did not understand the contingency is unclear (O’Neill et al. 2021).

The results of both studies indicate that, as in the majority of the string-pulling experiments, the tested parrots seemed to lack functional understanding of the presented trap and tube tasks.

#### Aesop’s fable

To test a causal understanding of water displacement in kea, Schwing et al. (2019) applied a widely used test paradigm known as the ‘Aesop’s fable’ (see review in Jelbert et al. 2015). This test involves placing stones into a tube of water to raise its surface level, allowing individuals to retrieve something floating on the surface of the water. The kea preferred to drop stones into tubes filled with water over tubes filled with sand. Additionally, they were able to apply what they had learned to a task variation that was functionally the same but perceptually different in that the water of the tubes was colored green, and thus appeared less transparent. However, the authors acknowledged that these tests might be insufficient to fully rule out a learned association of dropping stones in water-filled tubes to retrieve a reward. Therefore, they applied another variation of the set-up where two tubes were again filled with water but had holes from which the water could run out when the water level was raised. Crucially the holes were positioned at different heights of the tube rendering one unsolvable (the hole is positioned directly above the initial water level, thus water leaks as soon as stones are dropped) and one solvable (the hole is further up than the required water level). The kea were unable to consistently solve the leaking variation of the task, neither on an individual nor a group level. The findings of this study thereby also challenge the conclusions of earlier studies on causal understanding which did not use similar controls. Moreover, a line drawn at the water level for half of the birds to facilitate the perception of water level changes did not influence their performance. Whether kea did not pay attention to the crucial elements (the position of the hole) or could not truly understand the causality between the objects and the water levels in a confined space remains an open question (Schwing et al. 2019).

### Use of objects properties and physical entities

As already discussed, parrots explore the physical environment in a largely haptic manner (see e.g., Auersperg 2015; Le Covec et al. 2019). In this section, we summarize recent work related to the use of physical features of objects or physical entities.

When reproducing properties of a previously seen object (for example while sketching a house) we have to remember essential aspects of this object and might form a mental template to do so. Laumer et al. (2021a) tested whether Goffin’s cockatoos can recall and recreate previously rewarded cardboard strip templates in a replication of a study on New Caledonian Crows (*Corvus moneduloides*; Jelbert et al. 2018). Before each test, the subjects were trained to drop a piece of cardboard into a tube choosing either a particular color or length of strip or an L-shaped piece. In a color test, the correct color was rewarded. In tests for length and shape, the cockatoos were randomly rewarded to prevent trial-and-error learning. The birds reproduced the previous properties by either cutting out a strip from one of two differently colored large squares (color condition; all individuals successful) or by biting out a shorter or longer strip after being rewarded for longer or shorter templates (size condition, half of the birds successful), however they did not manufacture a shape after training on L-shaped pieces (shape condition). Whether this showed a cognitive limit or was due to morphological restrictions (biting around a corner) could not be determined. Overall, the Goffin’s showed similar results as the New Caledonian crows (which were not tested in a shape condition in Jelbert et al. 2018; Laumer et al. 2021a).

Little is known so far about parrot’s perception or understanding of weight. In a previous experiment investigating how information is acquired during object exploration, kea were able to distinguish between objects of a certain weight when paired with a distinct color but not with a possibly less salient feature of patterns (M. L. Lambert et al. 2017). In an effort to study weight discrimination in Goffin´s cockatoos, P. J. Lambert et al. (2021) provided them with a discrimination task. Half of the birds had to choose one of two balls that differed in both weight and color while the other half were provided only with weight information (the balls were otherwise identical). For both groups, either the light or heavy balls were rewarded. The first group reached the learning criterion faster than the second which had to solely rely on weight cues. In five subsequent sessions, all birds were only provided with weight cues, and all chose the ball with the correct weight significantly above chance level. Interestingly, individuals from the group that had previously learned with additional color cues performed better in the weight-only condition, suggesting that they were not solely using the color information to solve the task during training. In comparison to chimpanzees (Povinelli 2011), Goffin’s required strikingly fewer trials to differentiate between identical objects of different weights. Whether this was due to slight—but possibly important—differences in methodology or different ecological importance of attending to an object´s weight demands further investigation (P. J. Lambert et al. 2021).

To test whether budgerigars are able to match tones that resembled octave equivalence Wagner et al. (2019) trained birds to peck a response key for correct choices (go/no-go procedure). Four out twelve budgies learned the task contingencies successfully in training. However, in test trials they did not match their responses to octaves of the stimuli. This is surprising as budgerigars are a species known for their vocal mimicry and suggests that mimicry is not necessarily connected to the perception of octave equivalence (Wagner et al. 2019).

In a study on the understanding of the characteristics of liquids, Cornero et al. (2020) tested four African grey parrots on Piagetian liquid overconservation – the ability to keep track of (in this case) a quantity of juice in a cup, choosing the cup with the largest amount of juice. The basic procedure of the task was as follows: two initial cups filled with juice were presented to the subjects. Then the juice was poured into ‘destination cups’, and the parrots were allowed to choose one of these two cups. Depending on the condition, different information was available to the birds. The parrots were able to track the larger amount of juice even when the destination cups were opaque, and when the transfer was conducted in a crossed manner (e.g., liquid from the initial cup on the left side to the destination cup on the right side). Furthermore, they were not fooled by perceptual manipulation of the destination cups which made the amount of liquid appear to be equal in both cups. The parrots chose at random or adopted a side preference in the control tasks in which it was impossible to infer the correct location. In the last stage, only two birds were available for testing. Here, the destination cups were rigged in a way that made the lesser liquid appear to be of a larger quantity. One bird again chose the correct cup despite this misleading information while the second parrot chose correctly above chance in the trials with direct transfer (liquid from the left initial cup being poured into the left destination cup) but chose at chance when liquid was transferred in a crossed manner. A possible explanation might be the cognitive load involved in both tracking the liquids and the evaluation of misleading perceptual information (Cornero et al. 2020).

Bastos and Taylor (2019) set out to investigate whether kea could follow trajectories while simultaneously keeping track of the identity of two objects. Two tokens, one associated with a reward and one not, were shown to the birds and thereafter moved along different trajectories (see below) while being hidden inside the experimenters' fists. After the movement, the subjects could choose between the two hands, only one containing the rewarded token. In the control condition, both hands moved in parallel while being visible to the birds (tokens hidden in closed fists). In the crossed condition both hands disappeared behind an occluder but followed a straight line trajectory while the experimenter crossed their arms. Thus, the birds could observe the beginning and the end state of the movement. If the trajectory of the hands was not changed behind the barrier, it meant that the reward arrived at the opposite side from where it vanished. Note that the birds were trained in the parallel and crossed condition without an occluder before the start of the test. In the condition called ‘split’, the birds observed the same behavior but the experimenter switched the token behind the occluder. Therefore, the rewarded token emerged (invisible to the bird in the closed fist of the experimenter) at the same side it disappeared, thus “breaking” the assumed trajectory. The authors suggested that success in the parallel control condition (hands visible) and the crossed test condition (hands only visible at beginning and end), but chance performance or mostly incorrect choices in the split condition, would suggest a representation of invisible trajectories. Although the choices in the crossed and split conditions varied substantially between the individuals, four out of ten birds chose according to the pattern described above, and moved on to the next phase. This second experiment was implemented to test whether birds that had been successful in the crossed condition were following a simple heuristic of ‘choose the other side‘. Both hands were moved to the center of the screen and returned through a U-shaped trajectory at the side of the start position. Here only one side was occluded so the subject could see the movement and the trajectory of one hand. Three of the four tested kea chose the correct hand. A third experiment aimed to test their ability to predict where a reward-token would appear. The parrots had to choose between two occluded windows after seeing the initial start of the object's trajectory. Four out of seven birds did so above chance. In control conditions where conflicting information was given (one part of the occluder was removed and the birds could thus infer that the hand was not following the trajectory) all birds were able to choose the other window. The authors concluded from the study that some kea were able to simultaneously represent the trajectory and identity of the hidden objects (Bastos & Taylor 2019).

Previous studies have shown that kea can transfer learned discriminations from object to picture and vice versa (Wein et al. 2015), but find it easier to discriminate between real than digital objects in a reversal-learning task (O’Hara et al. 2015b). A recent study investigated whether kea can perceive the real and virtual world as continuous, i.e., that a virtually displayed process can have a real physical impact (Bastos et al. 2021c). Kea could observe a seesaw tilting to one side, which resulted in a token rolling off the seesaw and falling into one of two containers. They were trained to choose the correct side either with the seesaw being a physical object that dropped physical tokens into real occluded boxes (‘real condition’) or a digitally displayed seesaw with virtual tokens and boxes (‘virtual condition’). After reaching the criterion, the birds were presented with a ‘crossover’ condition: a virtual seesaw, virtual tokens but real occluded boxes. All kea chose the correct real box significantly above chance. When presented with the choice between a real token or a virtual token being dropped in a virtual box in follow-up trials, the kea showed no preference for either of the tokens. Half of the kea avoided virtual tokens when it seemed to touch but not fall into a real box (proximity control). The authors argued that this suggests that kea perceive virtual environments as equivalent and continuous with the physical world (Bastos et al. 2021c).

### Tool use

Several studies on tool use in parrots were published in recent years. We have already touched upon the self-care tooling innovation (see Fragaszy and Mangalam, 2018 for a discussion on tooling as a special form of tool use) of a kea (Bruce) who is missing his upper beak but instead substitutes it with a pebble during preening (Bastos et al. 2021a). To assess whether this behavior is deliberate, the authors evaluated Bruce´s use of pebbles over 20 h. They found that in the vast majority of cases Bruce picked up a pebble, he applied it to his body (93.75%). When he dropped a pebble during the process, he retrieved the same or a similar pebble to continue preening in 95.42% of cases and specifically selected pebbles of small size. Moreover, the stones chosen by Bruce for preening were considerably smaller than the stones chosen by other kea for object play and none of the other kea was observed using pebbles for preening (Bastos et al. 2021a).

As Goffin´s cockatoos are known to manufacture tools (Auersperg et al. 2012b), a recent study investigated how they adjust their tool manufacture to specific task demands (Auersperg et al. 2018). The cockatoos were presented with apparatuses that either required different lengths of tools or allowed for different widths. For both, they were trained on a ‘medium’ baseline distance/width and later presented with apparatuses that allowed for shorter and longer tools or wider and narrower tools than baseline. Although Goffin’s did not match the *exact* length needed for each task, they produced significantly longer tools when confronted with the reward at a larger distance than at a shorter one. Moreover, they often discarded tools that would have been too short before using them. Note that birds manufactured their tools by biting them out of cardboard, meaning that the investment increased gradually with the length of the tool. Goffin’s did not adjust the width of their tools to the width of the opening of the apparatus, and only one bird successfully retrieved the rewards when the width needed to be smaller than the trained baseline (see a discussion on the possible influence of her beak morphology; Auersperg et al. 2018).

Associative tool use describes the action of using two or more tools at the same time to achieve an end, irrespective of whether they are attached to one another or not. In the special case of composite tool use, multiple objects are combined to achieve a goal (see e.g., Shumaker et al. 2011; Wimpenny et al. 2009). Osuna-Mascaró et al. (2022) confronted subjects with an apparatus that had two pockets on each side. Each pocket held a platform and once something heavy was pushed into the pocket, the platform collapsed. One of the two platforms was baited. Therefore, the birds had to find a way to collapse the correct platform. They were provided with a stick and a ball. To solve the puzzle the birds first needed to insert the ball, then insert the stick and use it to push the ball to the pocket with the reward, i.e., combing both functions to collapse the platform. Five out of eight Goffin’s successfully solved the task during the experiment of which three did so above the previously set criterion of nine consecutive successful trials. One bird solved the task in the first trial and drastically reduced the time needed by the fifth. Notably, the three consistently successful birds used different insertion techniques (Osuna-Mascaró et al. 2022).

O’Hara and Mioduszewska et al. (2021) were able to observe two short-term captive but otherwise wild cockatoos, using sticks to open a sea mango stone. After thorough analyses, it became apparent that the cockatoos manufactured three distinct types of tools out of wood, which each had different physical properties and functions. As (up to) all three distinct tools were used to achieve a single goal – reaching the seed matter – this constitutes a tool set. Sturdy tools were used to wedge into a dorsal slot on the fruit stone and fine tools were used in a vertical manner to pierce an outer coating encapsulating the seedling inside the stone. Medium tools were used in both a vertical and a horizontal manner to lever out the seed matter. The study presents the first observation of the manufacture and use of tool sets in non-primates. Notably, 6 additional individuals of the group of 15 interacted with the fruit and combined vegetation but were unable to manufacture and successfully use tools. This suggests Goffin´s cockatoos are not habitually tooling on a species-wide level but that such behaviors result from individual innovation paired with opportunity (O’Hara and Mioduszewska et al. 2021).

### Concluding remarks physical cognition research

To summarize recent studies on physical cognition: Although parrots were able to solve a variety of physical problem-solving tasks, they largely failed the crucial controls for understanding causality (see sections ‘string-pulling task’, ‘Trap and tube problems’ and ‘Aesop’s fable’). String-pulling studies are frequent adopted, but many have used different variants to test specific questions and therefore results are difficult to compare. A higher level of standardization of methodologies across a great number of species might create better transparency in the future. However, the species recently tested in the condition with crossed strings, failed, which is in line with most – but not all – previously tested species. Studies on the use of object properties and physical entities show for example that some parrots form mental templates of some object properties (Laumer et al. 2021a), are sensitive to liquid overconservation (Cornero et al. 2020), discriminate based on weight (P. J. Lambert et al. 2021), and may perceive the virtual and real world as a continuum (Bastos et al. 2021c). Tool use studies in recent years included new coincidental observations that were further thoroughly examined (O’Hara and Mioduszewska et al. 2021; Bastos et al. 2021a). Moreover, an increasing number of studies show that some parrots can innovate not only tool manufacture, but also forms of associative tool use that were believed to be limited to primates and specialized tool users such as the New Caledonian crow (O’Hara and Mioduszewska et al. 2021; Osuna-Mascaró et al. 2022).

## Social cognition

Although parrots are often considered to have complex social hierarchies, in the wild, systematic research on their social systems and dynamics is rare (but see e.g., Hobson et al. 2014). Similarly, there was an imbalance in studies on parrot cognition, with an underrepresentation of studies on social cognition relative to studies on physical cognition. Areas which received the highest research effort in the social domain in the past include vocal communication (individual vocal learning, recognizing, matching calls to other individuals or groups) and social learning mechanisms (see M. L. Lambert et al. 2018 for review). Whereas, until recently, areas such as prosociality and inequity aversion remained largely untouched (see Heaney et al. 2017a; Péron et al. 2013, 2014 for exceptions). Nevertheless, in the past 4 years, these gaps have started to be filled.

### Self-recognition

One aspect of social cognition concerns the question of whether individuals are able to recognize themselves as distinct from others – an ability believed to be important for perspective-taking or theory of mind. The most common benchmark test for self-recognition is the ‘mirror self-recognition test’. Subjects are marked on a body part not visible to themselves (usually the head). If the subject then reaches to remove the mark quickly after seeing itself in the mirror, it is assumed to have understood that it is observing itself in a mirror, thus showing evidence of self-recognition. However this assumption is highly controversial (see e.g., Anderson and Gallup 2015; De Veer and van den Bos 1999 for a critical discussion on the test paradigm). Though kea and Goffin´s cockatoos removed their marks from a ventral area, they failed to do so when marked in other areas (van Buuren et al. 2019). Additionally, no increase in self-directed behavior could be observed when individuals of either species stood in front of the mirror. The authors concluded that neither species showed evidence of self-recognition (van Buuren et al. 2019).

### Cooperation

Previous studies have shown that kea are able to cooperate to some degree in a ‘loose-string’ test paradigm (Heaney et al. 2017b; Schwing et al. 2016). In this task, two individuals have to pull simultaneously on a string in order to bring forward a platform holding rewards. This needs to be done in a coordinated manner, i.e., if one subject pulls while the other does not, the reward is lost. Interestingly, two studies on kea showed strikingly different levels of successful cooperative behavior (18.9% in Schwing et al. 2016 and above 83% on average in Heaney et al. 2017b). This was particularly surprising considering the similarity in methodology between the two studies, prompting another study by Schwing et al. (2020). Results of this study suggest that pre-test training protocols may have played a key role in these differences. Indeed, in one of the studies, the kea were not required to pay close attention to the timing of the partner, as they had learned the task contingencies with a human experimenter always holding and pulling the string with the kea (Schwing et al. 2016). Whereas during the other study kea gradually learned that both ends needed to be held simultaneously in individual experience with the task (Heaney et al. 2017b). In a follow-up study, the group of the kea from Schwing et al. 2016 received more training to increase their attention to the human experimenter prior to the cooperation tests with conspecifics (Schwing et al. 2020). The experimenter waited for a set amount of time (0, 2, 4 or 6 s), picked up the string and held it for two seconds before putting it down. The parrots therefore had to pay close attention to the experimenter. In addition, kea only advanced to the testing phase after meeting a criterion. These changes drastically changed their performance in the subsequent test. Once all six birds had reached the criterion, they successfully cooperated in the vast majority of trials with a sufficiently long rope (100% in 11 out of 14 dyads). When a shorter string was used, success decreased, but dyads were still more successful than in the initial study. Moreover, when birds were forced to wait for their partner (by means of a barrier placed in front of one bird), increases in delay time resulted in decreases in success. This effect was stronger with shorter strings than with longer strings. Whereas they mostly failed in all delay conditions with the short string, the parrots were still successful in more than 40% of the trials with the longest delay of 6 s when the long rope was available. Subsequently, a triadic test scheme was introduced in which one central bird had access to two apparatuses while two other birds had access to one each. Therefore, if the central parrot was to cooperate with both partners it had to do so in a sequential manner, meaning that the second cooperator had to wait. Interestingly, kea were able to wait much longer in this situation which may be attributed to a more socio-ecological relevant set-up (in contrast to forced delays; Schwing et al. 2020).

During a further experiment, kea were given the opportunity to cooperate in a group setting. After the more dominant kea learned that they had to refrain from displacing others to be successful, they were able cooperate in groups of up to four individuals to solve a task (Schwing et al. 2021).

In a different study on blue-throated macaws, subjects in a dyad were able to solve the loose-string task when simultaneously given a string by the experimenter in 73.75% of trials (Tassin de Montaigu et al. 2020). Interestingly, they were even more successful (87.22%) when they did not see their partner. This may have been an effect of learning, as the second condition was introduced later in the experiment. In contrast to the kea, blue-throated macaws were not able to wait for their partner, which might again be due to differences in training procedure. The parrots did not seem to attend to the necessity of the partner, and likely applied the rule of simply pulling once the string is available.

### Prosociality

Except for two experiments on a pair of African grey parrots (Péron et al. 2013, 2014), all existing studies on prosociality in parrots have been published over the last 4 years. In prosocial choice tasks, African grey parrots (Krasheninnikova et al. 2019b) and kea (Heaney et al. 2020) behaved prosocially, but did not seem to understand the corresponding contingencies. Moreover, African grey parrots helped conspecifics in a token exchange task whereas blue-throated macaws did not (Brucks and von Bayern 2020) and individual Goffin´s cockatoos provided tools for a partner to access a reward (Laumer et al. 2021b). We will first elaborate on the recent studies on instrumental helping before comparing finds of prosocial choice tasks to the early studies on African grey parrots (Péron et al. 2013, 2014).

Brucks and von Bayern (2020) positioned the African grey parrots and blue-throated macaws in a test compartment with available tokens. The parrots were pre-trained to exchange tokens for food. In all but one condition (motivation control), access to the experimenter was blocked, preventing the subject from exchanging the tokens. The birds could, however, pick up the token and place it into the adjacent test compartment which was either empty (non-social control) or occupied by a partner. When a partner was present, it could either exchange the token with the experimenter to receive a reward in view of the focal subject (test condition) or was blocked from exchanging with the experimenter (social control and motivational control condition). The roles whether a bird was the actor or the receiver, were switched after each trial. African grey parrots transferred significantly more tokens in the test condition than in the social and non-social control, while blue-throated macaws hardly ever transferred tokens regardless of the condition. African grey parrots further increased the number of transfers after receiving help. However, this was independent of condition, leading the authors to conclude that the parrots might have copied their partner´s actions, or indirectly signaled that they were good coalition partners (as of the ‘prosocial honest signaling hypothesis’; Gintis et al. 2001) rather than the behavior being guided by calculated reciprocity. While the recipient´s behavior did not change significantly between conditions, attention-seeking behavior generally resulted in more transfers. The authors discussed the species differences with regard to their socio-ecological background. As African grey parrots live in more dynamic, fission–fusion groups compared to blue-throated macaws, prosocial tendencies might be more valuable in their everyday life. This might be reflected in higher rates of food-sharing seen in captive African greys (Brucks and von Bayern 2020).

A study on Goffin’s cockatoos also examined prosociality by exploiting their ability to use tools with different functions (Laumer et al. 2021b). Two birds were separated into different sections of a cage by a plexiglass wall with two windows. On each side of the cage, birds had access to different aspects of a puzzle box. One individual had access to one of two possible apparatuses baited with a reward. The tool to access this reward, however, was not available to the bird. The second individual, the actor, had access to four different objects, each of which had served as tools in previous contexts. The actor could choose to pass any of these tools through a window to the other bird. Two of the available tools (stick and ball) were associated with the two possible apparatuses, and two of the tools were ‘useless’ objects (not applicable for the two apparatus in this experiment but served as tools in a former study: Habl and Auersperg 2017). For their partner to receive a reward, the actor had to choose the correct tool, and pass it to the receiver through a window. The receiver was then able to use it on the apparatus inside its compartment to obtain the reward. The study also implemented control conditions where either the partner or the apparatus and reward were missing. On a group level, transfers were not affected by condition. Neither was the number of correct tool transfers nor the initial correct tool transfers (it was possible to transfer multiple tools per session). However, the analysis revealed a large effect of subject. On an individual level, three actors transferred the correct tool more often in the test trials than in the no-partner control. One of the Goffin’s, mostly transferred the correct tool before any other tool in the test condition. The authors therefore concluded that instrumental helping was limited to individuals and discussed the results in light of the relationships within the dyads (Laumer et al. 2021b).

Other studies have investigated prosociality through token choice tasks. In two previous studies, two African grey parrots alternately had to choose tokens, representing four options: ‘giving’ (only the partner received a reward), ‘sharing’ (both get a reward), being ‘selfish’ (only the actor received a reward) or to decline altogether (‘null’; neither bird received a reward; Péron et al. 2013, 2014). One of the birds seemed to attend to the partner's choices and adjust its behavior to some extent. Krasheninnikova et al. (2019b) recently conducted a follow-up study in which parrots had to decide between a prosocial or selfish choice depending on the token´s color. For example, for one dyad exchanging a blue token with the experimenter resulted in a sunflower seed for both parrots (prosocial choice) whereas exchanging a grey token would only provide a reward for the actor. When the birds chose the prosocial token in the unequal condition the actor was rewarded with a sunflower seed and the recipient received a walnut (preferred over sunflower seed). In the experiment, either one bird was the sole actor (‘unilateral condition’), the roles were repeatedly switched (‘alternating condition’), or the experimenter copied the choices of the partner bird from each trial of the previous alternating session (‘yoked control’). In control trials without a partner present, the parrots were either able to access the adjacent compartment and consume both rewards (‘accessible condition’) or not (‘inaccessible condition’). Lastly, a social facilitation control was implemented where a partner was present but not able to receive a reward from the experimenter.

When the rewards were equal, the parrots most often chose the prosocial token in the alternating and yoked conditions and, furthermore, prosocial choices were contingent on the previous choices in the alternating condition. These results support previous studies (Péron et al. 2013, 2014). However, in the present study, no difference in prosocial choices was found between the social control condition and when one bird was the sole actor (unilateral condition). Moreover, the birds chose the prosocial token more often in the inaccessible condition than accessible, unilateral, and social facilitation control, although they decreased prosocial choices in the inaccessible condition over time. Taken together, the results showed seemingly prosocial and reciprocal behaviors, but the prosocial choices in control conditions suggest that the subjects did not fully understand the contingencies of the task. The influence of payoff (equal or unequal) seemed to be dependent on the condition (Krasheninnikova et al. 2019b). We will discuss the results for unequal payoffs in the section ‘inequity aversion’ (see below).

A similar, yet slightly simplified, version of this study was conducted on kea (Heaney et al. 2020). The subject also received unilateral, alternating, yoked, and additionally non-social conditions. The payoff of prosocial choices was always equal. On a group level, prosocial choices occurred most often in the alternating, yoked, and non-social conditions. The authors argued that the general preference for the prosocial token could have resulted from a positive response to seeing multiple rewards, irrespective of whether the subject received both rewards, or just one. The same picture emerged on an individual level, with one bird showing a tentative pattern of making fewer prosocial choices when the partner was absent in the latter part of the study. Contrary to predictions, a correlation revealed that the parrots more often chose the selfish token after their partner chose prosocially in the previous trial (Heaney et al. 2020).

These studies have reported a tendency for different degrees of prosocial behavior in some, but not all, parrot species. Some parrots exerted effort to help conspecifics (Brucks and von Bayern 2020; Laumer et al. 2021b), whereas others provisioned partners at no cost to themselves (Krasheninnikova et al. 2019b; Heaney et al. 2020). However, when no effort was necessary, birds also often chose to be prosocial in control conditions, in which the prosocial choice did not have any effect on the partner.

### Inequity aversion

Until recently, it was not known to what extent parrots were sensitive to unequal payoffs. While kea were less cooperative in the loose string paradigm when the partner took more than its fair share of food (Schwing et al. 2016), they did not change their behavior in a token exchange paradigm when partners were equally rewarded for less work effort or with unequal payoff for the same effort (Heaney et al. 2017a).

Returning to the prosocial choice task on African grey parrots described previously (Krasheninnikova et al. 2019b), the birds choices were to some extent contingent on the parter´s choice in the alternating condition – even more so when rewarded unequally than equallly. Interestingly, they more often acted prosocially in the unequally rewarded unilateral condition than when they were equally rewarded. A possible reason for this may be that the subject seeing the favored reward (even though it was delivered to the recipient) increased the preference for the prosocial token. In contrast, they less often chose prosocially in the unequal alternating condition than when equally rewarded. However, over the course of the experiment they increased prosocial choices in this condition. In summary, the African grey parrots did behave differently when unequally rewarded, but the results are not consistent with inequity aversion (Krasheninnikova et al. 2019b).

In a different study, Krasheninnikova et al. (2019c) tested African grey parrots, blue-throated macaws, great green macaws and blue-headed macaws with a task in which both birds of a dyad had to exchange a token with the experimenter. In the equal conditions they were then rewarded the same (both received either low- or high-quality rewards). In unequal conditions the focal bird either had to invest more effort (the tokens had to be exchanged twice) or was rewarded less than the partner (the partner received a walnut). In control conditions the partner was not present. Compared to the equal conditions, African grey parrots and blue-headed macaws exchanged the token as often in the unequal conditions, while great green macaws exchanged less and blue-throated macaws generally exchanged less for lower-quality rewards. However, great green macaws also exchanged tokens less often when the walnut was delivered to an empty compartment (control condition). Considering this, overall, the parrots did not show an aversion to the partner receiving better food for the same effort or the same food for less effort (Krasheninnikova et al. 2019c).

A similar study was conducted more recently by Laumer et al. (2020) on Goffin’s cockatoos. In four different conditions, the focal parrot always received a sunflower seed for exchanging a token. The second bird either (a) also received the same reward (equity condition), (b) received a higher-quality reward for the same action (inequity condition), or (c) received the sunflower seed without an exchange (‘free gift’ condition). To control for reward movement, a ‘non-social' condition (d) was included, in which no partner was present but the experimenters moved a high-quality reward toward the empty partner´s compartment. In all four of these conditions, Goffin’s could either reach the token from the back of their compartment, or could rake it out from within a tube (increased work effort). While the birds generally willingly exchanged tokens throughout the experiment, the likelihood significantly decreased with additional work effort (raking in the token before the exchange). Moreover, there were fewer exchanges by the focal subject when their partner received the sunflower seed for free than when the partner also had to solve a tool use task for its token. Goffin’s exchanged at similar rates when the partner received a higher-value reward for the same work effort (exchanging of tokens). In a follow-up experiment, designed to increase differences in payoff, the subjects started to refuse to participate from the second session onwards when they did not receive any rewards. This behavior does not necessarily show aversion to inequality but could also indicate a loss of motivation or a refusal to carry out unrewarded work in general as discussed by the authors (Laumer et al. 2020).

### Social learning and transmission

To investigate whether wild peach-fronted conures pay attention to vocal interactions of unknown conspecifics, Thomsen et al. (2021) conducted a playback study in the field. Calls of two different individuals were played shortly after each other. The call initiator took the role of the ‘leader’, while the second followed up with calls, hence the ‘follower’. The calls were repeatedly played, modified to gradually increase in synchronization. Then, two different speakers were placed further apart, and both of the original, unmanipulated calls were played back (but not in reaction to one another). The authors recorded all call responses from the adjacent group as well as any approaching behaviors towards the speakers. Overall, the conures chose to follow the leader more often (67% of all following events) than the vocal follower. Larger flocks responded with fewer calls in general, and response call rates were influenced by vocal role (leader or follower), as well as sex of the playback caller. Flocks called more often when they only partially followed the playback calls. Furthermore, contact call responses during the choice phase matched the leader more often and were more similarly matched when all individuals in the flock landed close to the speaker. The authors therefore concluded that the members of the flock were eavesdropping on the (artificial) interaction of the playback stimuli, and used this information for later choices (Thomsen et al. 2021).

We previously described the bin opening behavior of sulphur-crested cockatoos (Klump et al. 2021). Through an online survey over 2 years, the authors were able to conduct a spatial network analysis to track the geographical spread of bin opening behavior over time. They further identified individual differences, but also strong effects of geographic distribution. The increase in behavioral differences, together with the increase in spatial distance suggests that local subcultures were formed, providing the first evidence of emerging cultural trends in a parrot species.

### Concluding remarks on social cognition research

Recent studies on kea showed that they have the potential to coordinate with a least four partners when they have learned the task contingencies beforehand (Schwing et al. 2020, 2021), while blue-throated macaws, without such training, seem to be able to learn the basic task but fail the critical control (partner delay; Tassin de Montaigu et al. 2020).

Studies on prosocial behavior seemed to have been in limbo but have now made a comeback with four new studies in recent years. The pattern that is emerging is that parrots show prosocial tendencies in instrumental helping tasks (Brucks and von Bayern 2020; Laumer et al. 2021b). Whilst parrots tend to also choose prosocially in choice tasks, all species tested so far fail critical controls, suggesting that they have only a limited understanding of the task contingencies (Krasheninnikova et al. 2019b; Heaney et al. 2020). As the instrumental helping tasks and prosocial choice tasks differ with respect to cost and reward for the actor, future studies might want to incorporate designs testing the continuum between prosocial (no or low cost for the actor) and altruistic behavior (substantial cost).

Some of the first studies on inequity aversion have suggested that this may not be a strongly expressed trait in the species tested so far. While Goffin’s cockatoos showed some aversion to unequal work effort, there was no evidence of aversion towards unequal rewarding in neither cockatoos (Laumer et al. 2020) nor African grey parrots (Krasheninnikova et al. 2019b, c). An important next step would be to carry out further studies on other species, specifically targeting unequal work effort.

The past 4 years have seen fewer studies on social learning in parrots (but see Thomsen et al. 2021), despite this having been a focus in the past. Notably, however, the spread of a foraging innovation of sulphur-crested cockatoos has been tracked and discussed in detail, providing evidence of emerging cultural trends in parrots (Klump et al. 2021).

## The primate/parrot cognition test battery

One study touched upon both the physical and the social domain. In an effort to replicate the Primate Cognition Test Battery (Herrmann et al. 2007, 2010; Schmitt et al. 2012) on parrots, a research team at the `Comparative Cognition Research Station` on Tenerife tested 37 individuals of 4 species: 9 great green macaws, 12 blue-throated macaws, 8 blue-headed macaws, and 8 African grey parrots (Krasheninnikova et al. 2019a). Methodological deviations from the Primate Cognition Test Battery were kept to a minimum and were strictly limited to necessary changes due to the morphological differences between primates and birds. Fifteen tests were used to study cognitive abilities in the physical (space, quantities, causality) and social (social learning, communication, theory of mind) domains. The general idea was to provide a comparison between parrots and primates, as well as between different parrot species. Contrary to the same parrot species’ performances in previous studies, subjects often performed at chance level across the PCTB tasks and therein showed no significant differences between the species or the domains (although some individuals performed well in single tasks). When directly compared with primates, all of the parrot species performed similarly to chimpanzees (*Pan troglodytes*) in the tests within the social domain, but worse in tests within the physical domain. The authors discuss the implications and possible shortcomings of the overall battery design but one of these may be the ‘anthropocentric’ nature of the battery as it was originally developed to test hypotheses focusing on the evolution of human cognition. Another downfall was a possible age effect. The majority of birds tested (31 out of 37; all African grey parrots and all blue-headed macaws) were juveniles, confounding results as many large parrots have long developmental periods, reaching sexual maturity only after multiple years. Cognitive development may similarly take a long time to reach a mature level. A post-hoc comparison on age of great green and blue-throated macaws however did not support this explanation (Krasheninnikova et al. 2019a).

Findings on the PCTB in parrots are indicative of the challenge of producing fair direct comparisons between distantly related species, which are required to achieve a better understanding of cognitive evolution.

## Conclusions, trends, and tools

Research on psittacine cognition has developed into a vibrant field with over 50 new studies in the last 4 years alone. Some topics such as inhibition control or understanding causality have now been tested in a range of studies and different species, leaving us with a more-fine grained, yet increasingly complex, picture. In the past, psittacine research focused disproportionately more on physical cognition than social cognition (see M. L. Lambert et al. 2018) though this seems to be changing, with a recent burst of new studies focusing on the social domain. While recent research has provided us with a first glimpse of topics previously unchartered in parrot cognition research such as probabilistic reasoning or sophisticated forms of tool use, advances have also been made in understanding core fundamental processes such as working, short-term and long-term memory in parrots. Moreover, we found that replicating studies with slight variations of test procedure can provide us with important information regarding pitfalls of task designs and understanding underlying mechanisms (Schwing et al. 2020). In the review which we chose as our starting point, M. L. Lambert et al. (2018) discussed the overall lack of socio-ecological knowledge on parrots, and the issues that surround studies being largely based on only a handful of model species. While this remains true, considerable contributions have been made since. Whilst Goffin´s cockatoos have been tested in captivity for over a decade, only recently research in wild populations has provided much-needed insight into their natural ecology (Mioduszewska et al. 2018, 2022; O’Hara et al. 2018). Similarly, a recent study on sulphur-crested cockatoos provides some of the first systematic evidence of social networks and social complexity in wild parrots (Aplin et al. 2021; but see e.g., Hobson et al. 2014). As the highly diverse order of psittacines provides us with ample opportunities, we are optimistic that future work will provide us with further knowledge to address ultimate questions of proximate findings. Laboratory studies have so far largely focused on a few model species, most notably African grey parrots, kea, and Goffin´s cockatoos. However, several recent studies have also focused on a number of neotropical parrots, broadening our knowledge of this diverse order.

Apart from the aforementioned studies on the socio-ecology of parrots, one can observe an overall trend in cognitive research to conduct studies in the wild (as discussed e.g., in Rosati et al. 2022). Early studies on psittacine cognition in wild settings have been rather scarce (for exceptions see e.g., Berg et al. 2011, 2012; Gajdon et al. 2004, 2006). This may in part be due to the methodological challenges of a less controlled environments (but see e.g., Pritchard et al. 2016 for review and discussion on the implementation of experimental designs and alternatives in field studies), as well as the immense and long-term research and logistic effort often required for such studies. It is exceedingly difficult to track and observe individuals in their natural habitat due to their frequent and fast locomotion and their arboreal lifestyle which is often centered in the dense canopy of tropical rainforests (as discussed e.g., in Mioduszewska et al. 2022). Nonetheless, we can observe a trend in wild cognition in recent years (Heinsohn et al. 2017; Loepelt et al. 2016; Osuna-Mascaró and Auersperg 2018) with multiple studies within the 4 years covered by this review (Klump et al. 2021; O’Hara and Mioduszewska et al. 2021; Thomsen et al. 2021).

A previously popular tool for conducting controlled studies on wild animals is a capture-release procedure, in which animals are caught for a limited period of time, tested – often under similarly controlled conditions as standard lab experiments – and released back into their natural habitat (to name a few: Cole & Quinn, 2012; Rössler and Mioduszewska et al. 2020; Tebbich et al. 2010; Webster & Lefebvre, 2001). Such studies, however, need to be ethically considered and carefully controlled by local experts to allow the birds to successfully re-socialize and continue to forage efficiently upon release.

Intriguingly, in a recent theoretical paper, Horn et al. (2022) proposed to abandon the strict ‘dichotomy between field and lab’, replacing it with a focus on the complexity of interdependent influences in variable contexts. For example, some possibilities along the field-lab continuum could range from parrots living in their natural habitat, largely undisturbed by anthropogenic influences, in highly urbanized areas or enriched group aviaries, or being kept as pets. The circumstances parrots live with, and thus the possible influences those conditions have on the expression of behavior and cognitive abilities, vary along multiple such continua (another example could be social structure of specific populations or context of testing). Furthermore, invasive parrot species living in highly urbanized areas can function as ‘wild labs’ to directly observe how species adjust to new challenges (see e.g., Klump et al. 2021; Osuna-Mascaró & Auersperg 2018).

However, the small number of parrot species tested in different contexts prevents any fine-grained and generalizable conclusions to be made as of yet (but see possibilities of ‘big-team research’ below). So far, only few experiments have addressed questions on populations of the same parrot species in different living conditions (Godinho et al. 2020; Rössler and Mioduszewska et al. 2020).

Technological developments and the use of human societal trends (e.g., social media) are being utilized by research groups in this field, presenting new opportunities, especially for field studies. For example, tracking devices such as GPS used to tag individuals are getting increasingly smaller and cheaper (see e.g. Wild et al. 2022), thereby increasing opportunities for lesser funded research groups, investigation of species with smaller body sizes, or studies that combine different approaches. A shift towards greater transparency and open publishing by the scientific community (in part induced by the replicability crisis) also results in a surge of widely available resources. New, openly available tools are emerging which bear great promise for field research (see e.g., Cauchoix et al. 2022; Wild et al. 2022). Furthermore, researchers increasingly involve the interested public in their studies, in so-called ‘citizen science’ projects. Approaches include specially designed apps, the use of social media platforms, or content shared on publicly available sites such as video platforms (for critical review on citizen science see e.g., Dickinson et al. 2010).

An impressive example combining many of these developments is the study of sulphur-crested cockatoos in Australia. Since 2011 researchers have been marking individuals with wing-tags which can be identified by citizen science volunteers using a specifically designed mobile app (‘Wingtags’) and a social media group (Davis et al. 2017; Kirksey et al. 2018). Moreover, data collected by citizen researchers were validated using GPS loggers, providing insights into long-term social networks in these populations (Aplin et al. 2021). Through this system, it was possible to track an emerging innovation in these cockatoos – their bin-opening behavior—as well as the cultural transmission thereof (Klump et al. 2021).

Another interesting recent trend is big team research projects (see review in e.g., Coles et al. 2022). One organizational unit, usually consisting of people from different labs with similar interests, can set up a platform and coordinate wide comparative studies, pooling research investment and resources. A recently launched big team project which has the potential to advance comparative research in birds including parrots, is the ManyBirds project (M. L. Lambert et al. 2022).

Each of these developments brings its own set of challenges and, though they cannot substitute conventional research methods, they are sure to be valuable additions to our toolset for the study of the parrot mind.

## Data Availability

Not applicable.
